# Relationship between the upregulation of Notch1 signaling and the clinical characteristics of patients with papillary thyroid carcinoma in East Asia: a systematic review and meta-analysis

**DOI:** 10.1186/s12935-018-0723-8

**Published:** 2019-01-03

**Authors:** Libing Yuan, Lei Ma, Haibo Xue, Shoujun Song

**Affiliations:** 1 Department of Endocrinology and Metabolism, Binzhou Medical University Hospital, No. 661 Second Huanghe Road, Binzhou, 256603 People’s Republic of China; 2 Department of Dermatology, Binzhou Medical University Hospital, Binzhou, China

**Keywords:** Notch1 signaling pathway, Papillary thyroid carcinoma, Clinical characteristic, Meta-analysis

## Abstract

**Background:**

Many studies have aimed to clarify the relationship between Notch1 signaling and papillary thyroid carcinoma (PTC), but the results have been inconsistent to date. In the present study, a systematic review and meta-analysis were performed to analyze the relationship between Notch1 signaling and the clinical characteristics of PTC.

**Methods:**

Literature databases, including PubMed (Medline), Embase and China National Knowledge Infrastructure, were searched for relevant studies from inception to April 2018. A total of five studies, including 421 patients with PTC from China and South Korea, were included in the meta-analysis.

**Results:**

The results revealed that the upregulation of Notch1 signaling was positively correlated with lymph node metastasis in patients with PTC (OR = 3.25, 95% CI 1.14–9.23, P = 0.03). Additionally, positive correlations were found between Notch1 signaling and tumor size (OR = 4.34, 95% CI 1.66–11.38, P = 0.003), capsular invasion (OR = 3.49, 95% CI 1.90–6.41, P < 0.0001) and clinical stage of PTC (OR = 2.31, 95% CI 1.05–5.11, P = 0.04).

**Conclusions:**

The Notch1 signaling pathway may play a catalytic role in the progression of PTC, and upregulation of Notch1 signaling may have significant predictive value for the clinical prognosis of PTC.

## Background

Thyroid cancer is one of the most common malignant tumors of the endocrine organ and is the most common malignant head and neck tumor. The morbidity and mortality rates of thyroid cancer remain high among head and neck cancers and are increasing each year. Papillary carcinoma is one of the most common pathological patterns in thyroid cancer, accounting for 60%–80% of cases among Chinese adult patients and 79.3% of cases in the United States [1]. This pathological pattern is well differentiated and characterized by slow growth and low malignancy, but the disease has a multicentric occurrence tendency and may develop features such as lymphatic metastasis in the neck at an early stage [2].

With the advancement of molecular biology, the evidence for the close relationships between tumors and certain genes has grown increasingly abundant. The Notch protein family plays an important role in cell differentiation, organ development and tumor genesis, progression, invasion and metastasis [3, 4]. The Notch signaling pathway is highly conserved in the process of biological evolution, and its activation can both accelerate the genesis of cancer and restrain the genesis of cancer, which may be correlated with cell environment [5–9]. Additionally, Notch signaling plays the double roles of oncogene and anti-oncogene in human tumors. Notch-related genes were first identified as oncogenes in T-lymphocytic leukemia [10] and were abnormally expressed in multiple types of tumors in subsequent research. For example, depletion of Notch1 led to growth inhibition of ovarian cancer cells, suggesting its carcinogenic effect [11]. The activation of Notch signaling restrains the growth of prostatic cancer, small cell lung carcinoma, basal cell carcinoma and pancreatic carcinoid cells, suggesting its antitumor effects [12–15]. Apart from the process of cancer genesis, as one of the four main proteins of the Notch family, Notch1 signaling plays an important role in cancer development. Notch1 has been found in a higher state of activation in tumor cells from colorectal cancer patients with lymphatic metastasis or distant metastasis than in those at an early stage, and these cells have a higher invasion and metastasis ability [16]. Therefore, it was suggested that the activation of Notch1 signaling may play an important role in accelerating the progression and metastasis of tumors.

Human medullary thyroid carcinoma (MTC) tissue and TT cell strains of MTC could be restrained by the activation of Notch1 signaling by injecting doxycycline into mouse models of MTC, the mouse tumors demonstrated significantly slower growth than the normal control group [17]. Additionally, Notch1 had lower expression in a cell strain of follicular thyroid carcinoma (FTC), and the proliferation of cells could be restrained after transfecting the two types of cells with a plasmid expressing the Notch1 intracellular domain [18]. Therefore, Notch1 signaling could play an inhibitive role in the aforementioned two types of thyroid cancer. However, the relationship between the Notch1 signaling pathway and PTC, the most common type of malignant tumor of the endocrine organ, and the regulatory mechanism of its downstream target genes remain undefined.

The present meta-analysis, based on retrieved documents, was conducted to evaluate the correlations between Notch1 signaling and lymphatic metastasis and other clinical features of PTC, which may provide useful information for evidence-based medicine for the prognosis, prophylaxis and treatment of PTC.

## Methods

### Retrieval strategy

The research material was collected from published clinical case–control studies, with or without blinding. The language was limited to English and Chinese. Retrieval was conducted via a computer or retrospective database, such as PubMed (Medline), Embase and China National Knowledge Infrastructure (CNKI), from inception to April 2018. The retrieval words were papillary carcinoma of thyroid gland, papillary thyroid carcinoma, Notch1, Notch signaling, lymph node, thyroid cancer, lymphatic metastasis, tumor size, capsular invasion and clinical staging.

### Inclusion criteria

(1) One-off primary studies related to PTC, Notch signaling, lymphatic metastasis, tumor size, capsular invasion or clinical staging published and unpublished. (2) Patients with PTC staging criteria conforming to the 2015 thyroid cancer National Comprehensive Cancer Network (NCCN) guidelines. (3) The literature had raw data and provided the odds ratio (OR) and 95% confidence interval (CI), or these could be obtained from the data through calculation. (4) Original documents with objectives and complete pathological diagnosis. (5) No history of other malignant tumors.

### Exclusion criteria

(1) Documents with nonrigorous experiments and incorrect statistical approaches. (2) Repeatedly published thesis or research. (3) No control group. (4) Lacking an objective or complete pathological evidence.

### Expression of Notch1 signal

We defined high and low Notch1 expression by immunohistochemical staining evaluation, as well as through detailed reading of the full text, contacting the author and other methods of collecting information, and after strict discussion, we excluded articles that cannot be merged, such as those with (1) unqualified primary antibodies; (2) serious interpersonal bias; (3) unscientific positive definitions; (4) grouping methods that could not be merged.

### Data extraction and quality evaluation of included documents

Papers were retrieved and screened by reading their titles and abstracts, which was performed by two researchers independently. The abstracts were read to determine whether they met the criteria and eliminate unqualified literature. The information extracted included the name of the first author, year of publication, research design (e.g., sample size, randomization, allocation concealment, blinding method, pathological basis) and patient characteristics (expression of Notch1, lymph node metastasis, tumor size, capsular invasion and clinical staging). The quality of the selected papers was assessed by another two evaluators based on the Newcastle–Ottawa scale (NOS) [19]. The final score of the evaluation was the sum of the selection of the study group (4 points), the intergroup comparability (2 points) and the measurement of the exposure factors (3 points). If the opinions were inconsistent, the final score was determined by discussion, or the average value to one decimal place was the final score if agreement was not reached. Studies with a score of 0–4.5 points were classified as low quality, and those with a score of 5–9 were classified as high quality.

### Statistical analysis

The meta-analysis was conducted using RevMan5.3 (Review Manager, Nordic Cochrane Centre, Danmark). The OR was used to represent the selected enumeration data, and the 95% CI of each OR was calculated and used to draw a forest map, which was named the dominance ratio map, to demonstrate differences between the results. Heterogeneity was determined by the I^2^ value, and only low-heterogeneity (I^2^ ≤ 25%) and middle-heterogeneity data (25 < I^2^ ≤ 75%), not high-heterogeneity data (I^2^ > 75%), were used for the meta-analysis.

## Results

### Basic features of the included studies

**Fig. 1:**
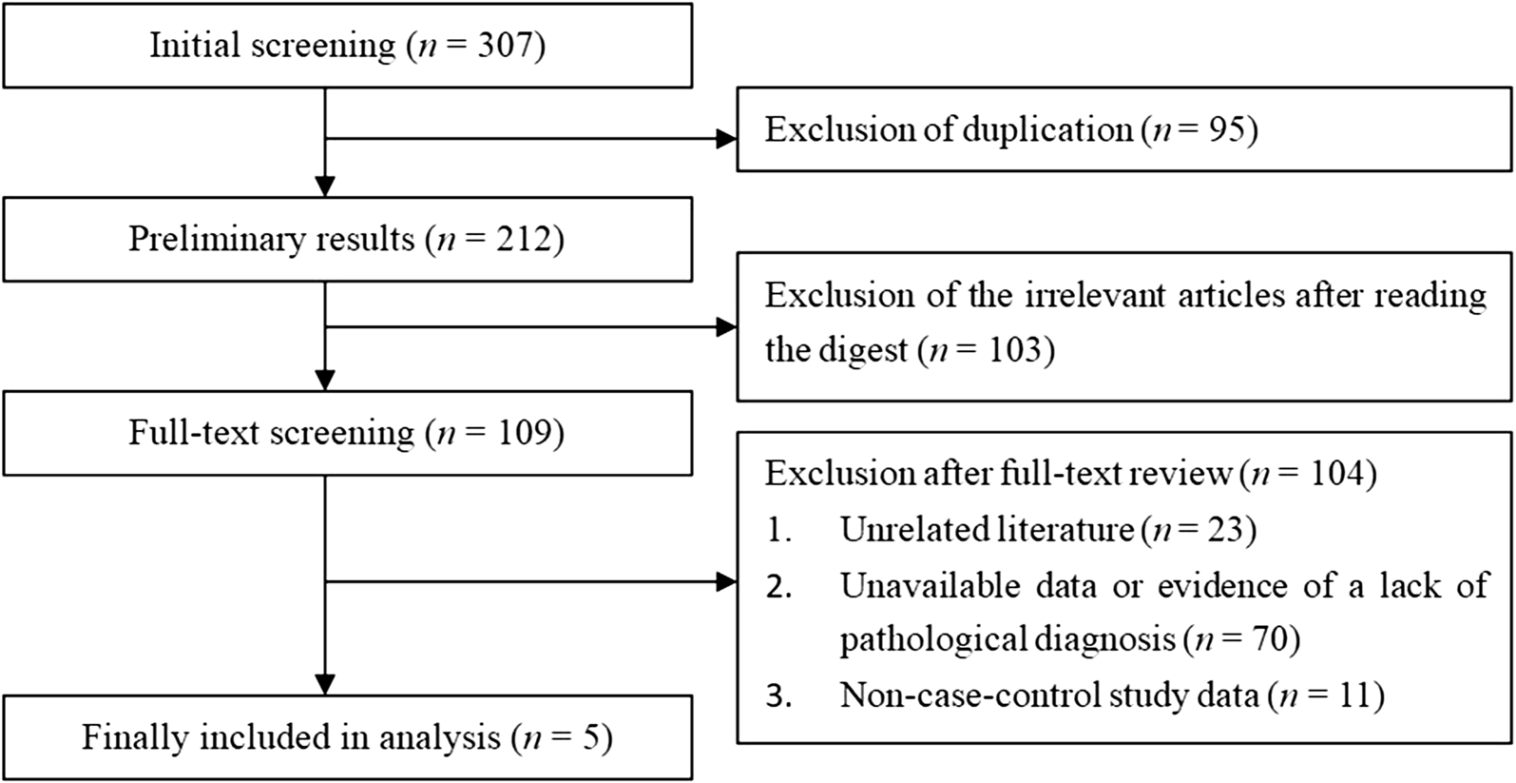
Literature screening process and results

After the initial screening, 307 documents were retrieved; 212 remained after removing duplicates; and 109 papers were selected through the initial screening after reviewing abstracts and prefaces. By reading through the full text, we eliminated 23 unrelated documents according to our inclusion and exclusion criteria. There were 70 papers without usable data or pathological diagnostic evidence and 11 studies without case controls. Finally, five studies were chosen, including three in English and two in Chinese [20–24]. All the cases included were from China and South Korea. The process of the systematic literature search is displayed in a flow diagram in Fig. 1. The characteristics and quality evaluation of the included literature are shown in Table 1.

**Table 1:** Characteristics and quality evaluation of the included studies

Study (year)	Case (*n*)	Country	Antibody for Notch1	NOS score
H. Fu (2016) [20]	68	China	Cell Signaling Technology, USA	7.5
H.S. Park (2012) [22]	174	South Korea	Abcam, UK	8.5
J. Zhang (2014) [23]	70	China	Epitomics, USA	6
Q. Long (2012) [24]	40	China	Santa Cruz, USA	6.5
M. Jing-You (2017) [25]	69	China	Boosen Biotechnology, CHN	7

Antibody for Notch1: Manufacturer of Notch1 primary antibody

*NOS score* Newcastle–Ottawa scale score

### Results of meta-analysis and heterogeneity analysis

**Fig. 2:**
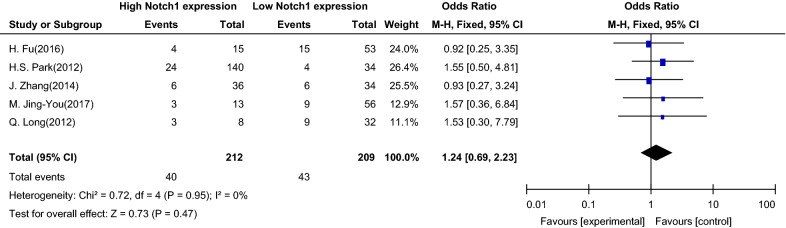
Association between Notch1 signaling and sex of patients with papillary thyroid carcinoma. *OR* odds ratio, *CI* confidence interval, *Event* male

In the five studies, the association between Notch1 signaling and sex of patients with PTC was analyzed, and an OR of 1.24 (95% CI 0.69–2.23) was found (Z = 0.73, P = 0.47 in the test for overall effect). These data reveal that there was no significant correlation between Notch1 signaling and sex in PTC patients (see Fig. 2 for details).

**Fig. 3:**
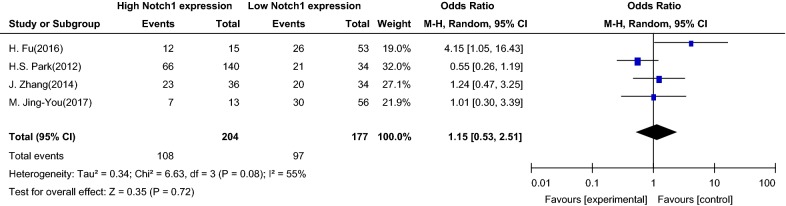
Association between Notch1 signaling and age of patients with papillary thyroid carcinoma. *OR* odds ratio, *CI* confidence interval, *Event* more than 45 years old

A total of 381 patients from five studies were analyzed to assess the relationship between Notch1 signaling and the age of patients with PTC, which was divided into two groups (≥ 45 and < 45). There was no significant association between Notch1 signaling and age (OR = 1.15, 95% CI 0.53–2.51, Z = 0.35, P = 0.72) (Fig. 3). Thus, the age-related relationship between Notch1 signal and PTC has not been clarified.

**Fig. 4:**
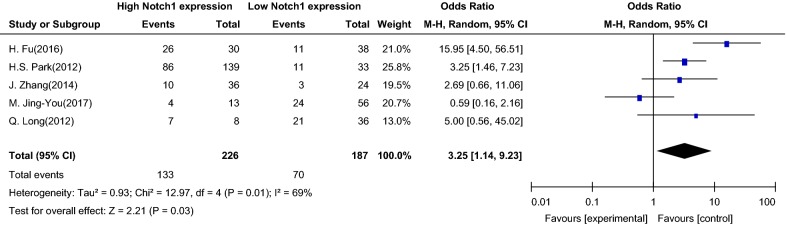
Association between Notch1 signaling and lymph node metastasis in patients with papillary thyroid carcinoma. *OR* odds ratio, *CI* confidence interval, *Event* patients with lymph node metastases

The meta-analysis of the association between Notch1 signaling and lymphatic metastasis of PTC in the five studies indicated that the OR was 3.25 (95% CI 1.14–9.23), and the 95% CI of the OR fell to the right side of the vertical line in the forest map (Z = 2.21, P = 0.03 in the test for overall effect), which suggested that there was a significant correlation between Notch1 signaling and lymphatic metastasis of PTC. Therefore, Notch1 signal overexpression may accelerate lymphatic metastasis of PTC (Fig. 4).

**Fig. 5:**
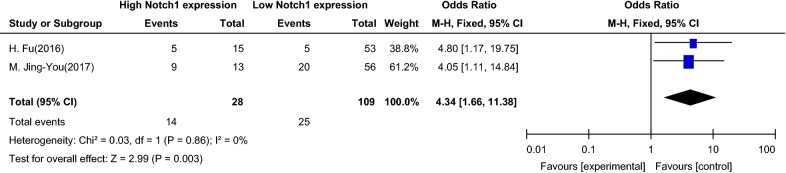
Association between Notch1 signaling and the primary lesion size of patients with papillary thyroid carcinoma. *OR* odds ratio, *CI* confidence interval, *Event* tumor diameter greater than 2 cm

Two studies were used to analyze the relationship between Notch1 signaling and the primary lesion size in patients with PTC. The patients were divided into ≤ 2 cm and > 2 cm groups according to the primary tumor size. The meta-analysis revealed an OR of 4.34 (95% CI 1.66–11.38) (Fig. 5). In the forest map, the 95% CI of the OR was on the right side of the vertical line (Z = 2.99, P = 0.003 in the overall effect test), which indicated that upregulation of Notch1 signaling may promote tumor enlargement in PTC.

**Fig. 6:**
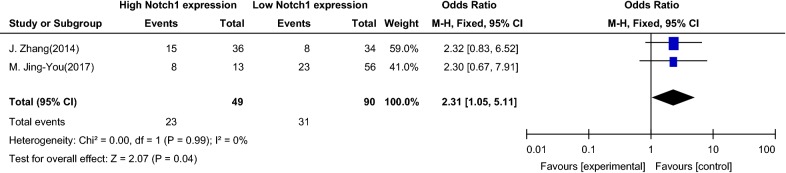
Association between Notch1 signaling and the clinical stage of patients with papillary thyroid carcinoma. *OR* odds ratio, *CI* confidence interval, *Event* stage I and stage II patients

The meta-analysis of the relationship between Notch1 signaling and the clinical stage of patients with PTC in the present study was based on two articles. According to clinical stage, patients were divided into two groups (one group with stage I and II, the other group with stage III and IV). Figure 6 shows an OR = 2.31 (95% CI 1.05–5.11), and the 95% CI of the OR was on the right side of the vertical line in the forest map (Z = 2.07, P = 0.04). These data suggest that overexpression of Notch1 may promote the invasion, expansion or metastasis of PTC.

**Fig. 7:**
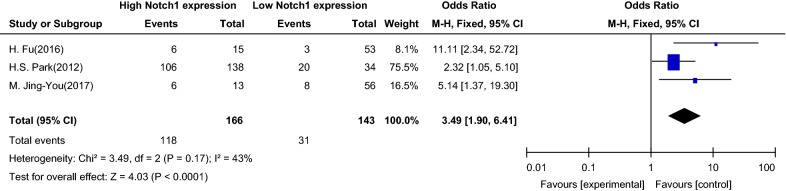
Association between Notch1 signaling and capsular invasion in patients with papillary thyroid carcinoma. *OR* odds ratio, *CI* confidence interval, *Event* patients with capsular infiltration

A total of 309 patients from three articles were analyzed to assess the association between Notch1 signaling and capsular invasion of PTC. The OR was 3.49 (95% CI 1.90–6.41), and the 95% CI of this OR fell to the right side of the vertical line in the forest map (Z = 4.03, P < 0.0001 in the test for overall effect). These findings demonstrate that upregulation of the Notch1 signaling pathway may contribute to the process of primary tumor capsular invasion in PTC (Fig. 7).

## Discussion

The Notch signaling pathway is involved in not only the normal development of the nervous and endocrine systems but also tumorigenesis in multiple tissues, including hematologic, breast, prostate, cervix, lung, liver, and brain malignancies [25–34]. In thyroid tumors, most studies on the role of Notch1 signaling have mainly investigated MTC because of the association between Notch expression and neuroendocrine development. Although PTC is the most common subtype of thyroid carcinoma, studies on the association between Notch1 signaling and its clinicopathological factors are limited.

As the most important member of the Notch family, the role of the Notch1 signaling pathway in the development of PTC is intriguing. The TNM system is the most widely adopted system for grading PTC, and large tumor size, lymph node metastasis, capsule invasion, and old age are high-risk clinicopathological characteristics in PTC. Because patients with these high-risk variables are prone to recurrence or metastasis even after operations, they are usually advised to undergo radioiodine remnant ablation (RRA) according to guidelines [35]. Therefore, we conducted the present study to determine whether there were some associations of clinical significance with the expression of Notch1 and these high-risk clinicopathological characteristics.

We pooled five studies and obtained a sample size of 421 cases of PTC in East Asia to yield more robust results. A systematic review and meta-analysis revealed that there was a significant positive correlation between lymphatic metastasis of PTC and activity of the Notch1 signaling pathway. Meanwhile, the trend of the relationship was highly consistent among the included studies, and four studies showed an estimated MHOR point of more than one. Therefore, we speculate that the activation of Notch1 signaling may promote the metastasis of PTC cells to lymph nodes. Grouping analysis was used to further evaluate the correlation between Notch1 signaling and other clinical features of PTC and revealed that Notch1 signaling was closely correlated with tumor size, capsule invasion and clinical stage in patients with PTC. Importantly, almost all the studies included in this meta-analysis suggested that higher Notch1 signaling would lead to poor prognosis in patients with PTC. The relationship trends in this analysis were highly consistent as well, and all the included studies showed a MHOR point estimate of more than one. Some previous studies reported that the downregulation of Notch1 signaling could reduce the proliferation of PTC cells [36]. Another recent study demonstrated that the activation of Notch1 signaling could promote the induction of epithelial–mesenchymal transition (EMT) and the progression of PTC [37]. These findings combined with the results of our meta-analysis suggest that upregulation of Notch1 signaling may be a valuable predictor of greater tumor diameter, higher risk of capsule infiltration and higher clinical stage in patients with PTC.

Although the incidence of PTC was higher among females according to the SEER investigation [38], we found no significant differences in the distribution of men and women among the patients with upregulated Notch1 signaling. Additionally, the patient’s age is considered an important variable for the diagnosis of Differentiated thyroid carcinoma (DTC) according to the TNM system, but we did not find any significant relationship between Notch1 signaling and age in patients with PTC in the present study, which further indicated that advanced age may be an independent risk factor for the prognosis of PTC. Moreover, it was reported that upregulation of Notch1 was involved in the chemotherapy resistance and tumor recurrence in glioblastoma [39]. Another study revealed that upregulation of Notch1 signaling was related to a higher recurrence rate in patients with PTC [40]. However, due to the inconsistent treatment methods in different studies, as well as the nonuniform diagnostic criteria for recurrence, we did not further explore the association between Notch1 signaling and the recurrence of PTC in the present meta-analysis. In addition, all the patients included were from Asia (China and South Korea), which limits the generality of our conclusions to a certain extent. More studies are needed on the activity of Notch1 signaling in patients with PTC in Europe, USA and Africa.

In summary, the results of the present study reveal that the Notch1 signaling pathway may play a catalytic role in the progression of PTC, and the activity of Notch1 signaling may be a significant predictive marker for the prognosis of PTC. Therefore, it can be suggested that PTC patients with higher Notch1 signaling activity may tend to have more tumor invasiveness and can benefit from more active treatment (such as RRA).

At present, there has been no decisive breakthrough in the study of the Notch1 signaling pathway, but there are many recent studies supporting our view. In the process of tumor invasion, the epithelial–mesenchymal transition (EMT) occurs in some cells, and tumor cells lose cell–cell adhesion. EMT leads to tumor cytoskeletal rearrangement, reduced cell rigidity and cell–cell connectivity, supporting tumor metastasis and invasion. Studies have shown that Notch receptors can be linked to myosin, thereby affecting the mechanical properties and physical ability of cells [41]. In addition, Notch1 can reduce the expression of zinc finger E-box binding homeobox (ZEB) and vimentin by regulating miR-200b, thereby promoting tumor cell EMT [42]. During cell migration, F-actin aggregates at the front edge of invasive cells and pushes the cell membrane outward to form protrusions. The protrusions are anchored in the extracellular matrix by integrin family transmembrane receptors and are coupled with actin cytoskeleton by adhesion spot proteins. ADAM12 is an anoxic effector and is regulated by Notch1. At the same time, ADAM12 is also a heparan-binding EGF-like growth factor (HB-EGF) shedding enzyme. Hypoxia activates EGFR via HB-EGF. The signaling pathway promotes the formation of invasive pseudopods, that is, hypoxia can increase the expression of ADAM12 by activating Notch1, thereby promoting the formation of invasive pseudopods induced by HB-EGF shedding [43]. Tumor cells penetrate the extracellular matrix barrier and the basement membrane of the vascular wall, and permeation of the haemorrhagic vessel wall into the host microenvironment is inseparable from the degradation of extracellular matrix and basement membrane. Matrix metalloproteinases (MMPs) are important enzymes that degrade the extracellular matrix and play an important role in tumor invasion and metastasis. MMP-2 and MMP-9 are the most important factors in the MMP family in promoting tumor invasion and metastasis. Studies have shown that activation of the Notch1 signaling pathway significantly increases the expression of MMP-2 and MMP-9 in the MMP family [44]. Inhibition of Notch1 can significantly reduce the binding ability of NF-kappa B to DNA, and the expression and activity of MMP-2 and MMP-9 are also significantly inhibited, thus reducing the metastasis ability of cancer cells. MMP-2 and MMP-9 activation enhances invasive ability [44, 45]. These results suggest that cell–cell adhesion can influence downstream gene expression through Notch1 signaling, thus altering the mechanical properties of cancer cells.

Some pathological factors, such as histological subtype, serum thyroglobulin and several other protein levels, are important and may affect the progression of PTC [46]. In the present study, the systematic evaluation suggested that the Notch1 signaling pathway has high clinical significance in the prognostic evaluation of PTC. However, a meta-analysis cannot substitute for direct evidence obtained from large-scale and multicenter randomized-controlled trials (RCTs). Furthermore, in-depth research into specific molecular mechanisms is needed to provide reliable theoretical foundations for the Notch1 signaling involvement in the pathogenesis and progression of PTC, and such mechanisms may be a new target for clinical treatment and provide predictive value in patients with PTC.

## Conclusion

Here, we searched electronic databases for relevant studies, and enrolled 5 studies with a total of 421 patients for meta-analysis, drawing a conclusion that the Notch1 signaling pathway may play a catalytic role in the progression of PTC. Taken together, the results from our meta-analysis suggest that PTC patients with higher Notch1 signaling activity may tend to have more tumor invasiveness. More multi-center prospective cohorts are warranted to further validate the role of the Notch1 signaling in PTC.

## Abbreviations

PTC: papillary thyroid carcinoma
CNKI: China National Knowledge Infrastructure
MTC: medullary thyroid carcinoma
FTC: follicular thyroid carcinoma
NCCN: National Comprehensive Cancer Network
CI: confidence interval
NOS: Newcastle–Ottawa scale
OR: odds ratio
RRA: radioiodine remnant ablation
EMT: epithelial–mesenchymal transition
DTC: differentiated thyroid carcinoma
RCTs: randomized-controlled trials
